# Genetic variation at aryl hydrocarbon receptor (*AHR*) loci in populations of Atlantic killifish (*Fundulus heteroclitus*) inhabiting polluted and reference habitats

**DOI:** 10.1186/1471-2148-14-6

**Published:** 2014-01-14

**Authors:** Adam M Reitzel, Sibel I Karchner, Diana G Franks, Brad R Evans, Diane Nacci, Denise Champlin, Verónica M Vieira, Mark E Hahn

**Affiliations:** 1 Biology Department, Woods Hole Oceanographic Institution, 45 Water Street, Woods Hole, MA 02568, USA; 2 Department of Biology, University of North Carolina at Charlotte, Charlotte, NC 28223, USA; 3 NHEERL, Atlantic Ecology Division, U.S. Environmental Protection Agency, Narragansett, RI 02882, USA; 4 Department of Environmental Health, Boston University School of Public Health, Boston, MA 02118, USA; 5 Current address: Department of Genetics and Genomic Sciences, Mount Sinai School of Medicine, New York, NY 10029, USA; 6 Current address: University of California, Program in Public Health, Irvine, CA 92697, USA

**Keywords:** Local adaptation, Pollution, Molecular mechanism, Resistance, Tolerance, Convergent evolution, Population genetics

## Abstract

**Background:**

The non-migratory killifish *Fundulus heteroclitus* inhabits clean and polluted environments interspersed throughout its range along the Atlantic coast of North America. Several populations of this species have successfully adapted to environments contaminated with toxic aromatic hydrocarbon pollutants such as polychlorinated biphenyls (PCBs). Previous studies suggest that the mechanism of resistance to these and other “dioxin-like compounds” (DLCs) may involve reduced signaling through the aryl hydrocarbon receptor (AHR) pathway. Here we investigated gene diversity and evidence for positive selection at three *AHR*-related loci (*AHR1, AHR2, AHRR*) in *F. heteroclitus* by comparing alleles from seven locations ranging over 600 km along the northeastern US, including extremely polluted and reference estuaries, with a focus on New Bedford Harbor (MA, USA), a PCB Superfund site, and nearby reference sites.

**Results:**

We identified 98 single nucleotide polymorphisms within three AHR-related loci among all populations, including synonymous and nonsynonymous substitutions. Haplotype distributions were spatially segregated and F-statistics suggested strong population genetic structure at these loci, consistent with previous studies showing strong population genetic structure at other *F. heteroclitus* loci. Genetic diversity at these three loci was not significantly different in contaminated sites as compared to reference sites. However, for AHR2 the New Bedford Harbor population had significant F_ST_ values in comparison to the nearest reference populations. Tests for positive selection revealed ten nonsynonymous polymorphisms in AHR1 and four in AHR2. Four nonsynonymous SNPs in AHR1 and three in AHR2 showed large differences in base frequency between New Bedford Harbor and its reference site. Tests for isolation-by-distance revealed evidence for non-neutral change at the AHR2 locus.

**Conclusion:**

Together, these data suggest that *F. heteroclitus* populations in reference and polluted sites have similar genetic diversity, providing no evidence for strong genetic bottlenecks for populations in polluted locations. However, the data provide evidence for genetic differentiation among sites, selection at specific nucleotides in AHR1 and AHR2, and specific AHR2 SNPs and haplotypes that are associated with the PCB-resistant phenotype in the New Bedford Harbor population. The results suggest that AHRs, and especially AHR2, may be important, recurring targets for selection in local adaptation to dioxin-like aromatic hydrocarbon contaminants.

## Background

Understanding the molecular basis of adaptation to environmental change is an important goal in environmental biology. Animal populations adapt to a variety of natural environmental stressors through genetic and epigenetic changes that affect gene expression or protein structure and/or function. Anthropogenic stressors, including toxic chemicals, can also drive selection in natural populations. For example, evolved resistance of insects to the acute neurotoxicity of insecticides is well known and occurs through a variety of mechanisms involving reduced target site sensitivity or enhanced expression of proteins involved in biotransformation and excretion of the chemicals [1-3]. Thus, we have learned a great deal about adaptation to chemicals designed to be toxic to their target organisms. However, field examples are less frequent and adaptive mechanisms are not as well understood for broadly distributed industrial pollutants that produce unintended consequences in non-target organisms. Recent studies (reviewed in [4-6]) have provided strong evidence for adaptation of fish populations to aromatic hydrocarbons such as polychlorinated biphenyls (PCBs), polychlorinated dibenzo-*p*-dioxins (PCDDs), and polycyclic aromatic hydrocarbons (PAHs) that cause toxicity similar to that caused by 2,3,7,8-tetrachlorodibenzo-p-dioxin (TCDD). These “dioxin-like compounds” (DLCs) are capable of interfering with embryonic development and eliciting acute and chronic effects on reproduction, immune function, and other essential processes [7,8] with population-level consequences [9].

Populations of the non-migratory Atlantic killifish *Fundulus heteroclitus* that persist in highly contaminated environments may provide insight into the molecular mechanisms by which natural populations adapt to long-term, multi-generational exposure to DLCs. *F. heteroclitus* is widely used as an environmental model [10] for studying adaptations to natural environmental variables such as temperature [11,12] and evolved tolerance to anthropogenic chemicals [13]. Several distinct and geographically distant populations of *F. heteroclitus* inhabiting highly contaminated Superfund sites have been demonstrated to possess enhanced tolerance or resistance to one or more DLCs as compared to reference populations. The most well-studied populations are found in Superfund sites at Newark Bay, NJ (EPA ID: NJD980528996, contaminated with TCDD) [14-17], the Elizabeth River, VA (EPA ID: VAD990710410, contaminated with PAHs from creosote) [18-20], and the Acushnet River Estuary (EPA ID: MAD980731335, New Bedford Harbor (NBH), MA, contaminated with PCBs [21]) [22,23]. Adaptation also has been demonstrated in *F. heteroclitus* inhabiting more moderately contaminated sites [6,24].

The molecular mechanism(s) underlying the DLC resistance are not known for any of these populations. However, the characteristics of the resistant phenotype provide important clues, which can be illustrated using the NBH population as an example. First, killifish embryos from NBH are less sensitive to the developmental toxicity of 3,3′,4,4′,5-pentachlorobiphenyl (PCB-126) than embryos from a reference site [22]. Second, when exposed to DLCs, NBH larvae display poor inducibility of the well-known biomarker cytochrome P450 1A (CYP1A) [22]. Similar insensitivity to DLCs is found in adult killifish from NBH, in which the resistance to altered CYP1A gene expression occurs in all tissues and at the level of gene transcription [23]. Third, the altered sensitivity of NBH fish to the toxic and biochemical effects of DLCs is heritable through at least 2 generations, consistent with genetic adaptation rather than physiological acclimation [6,22,25]. Most of these phenotypic characteristics are shared among independent DLC-resistant populations, suggesting that similar mechanisms of DLC tolerance have evolved in parallel at multiple sites [6,26].

Theoretical considerations suggest that adaptation to extreme pollution such as that found in NBH and other contaminated sites is more likely to result from major gene effects rather than polygenic adaptation [27,28]. Killifish populations at NBH and other highly polluted sites experience strong selection intensity (exposure to PCB concentrations well above the LC50), exhibit a large phenotypic shift (differences in sensitivity of two orders of magnitude as compared to reference populations [22]), have large population sizes [29], and have gene flow from neighboring populations [30]—all features that favor adaptation via single genes with large effects [27,28,31]. Consistent with this, previous studies have shown that the PCB-resistant NBH population has similar levels of overall genetic diversity compared with nearby populations of PCB-sensitive fish [29,32,33]. In light of these theoretical and empirical considerations and in keeping with a desire to employ a mechanistic perspective [34], we have taken a candidate gene approach to investigate the molecular basis of adaptation to DLCs in killifish.

The most likely candidates for major genes affecting sensitivity to DLCs are those encoding proteins in the aryl hydrocarbon receptor (AHR)-dependent signaling pathway, the master regulator of responses to many of the most toxic DLCs, including TCDD and the PCBs with TCDD-like effects. The AHR is a ligand-activated transcription factor that exhibits high affinity for TCDD and other DLCs, regulates expression of a large set of genes in response to DLC exposure, and is required for TCDD or PCB toxicity in mammals [35,36] and fish [37,38]. Previously, we identified and cloned multiple components of the *F. heteroclitus* AHR pathway, including two AHR paralogs (AHR1, AHR2), an AHR nuclear translocator (ARNT2), and AHR repressor (AHRR) [39-42]. We hypothesized that allelic variation at one or more of these loci resulted in proteins with reduced sensitivity to activation by DLCs. This hypothesis is consistent with results from other vertebrate species, where differences in the AHR pathway underlie many of the differences among species, strains, or cell lines in the sensitivity to DLC effects [43-46]. In some mammalian systems, reduced sensitivity to AHR agonists results from allelic variation at the *AHR* locus [47-50].

**Figure 1:**
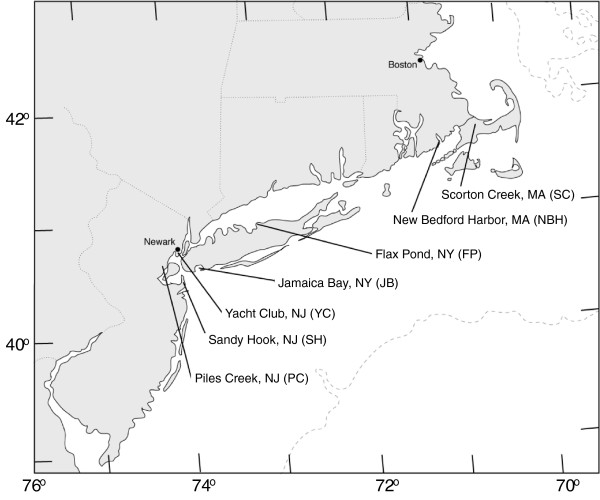
**Collection sites of *Fundulus heteroclitus *along the north Atlantic coast of the United States.** Sample sizes for each population are provided in Table 1.

To identify common genetic loci associated with tolerance, we compared variation in AHR-related genes within and among fish populations resident to highly contaminated sites and nearby, less-contaminated reference sites. In an earlier study, we identified multiple AHR1 alleles and observed differences in the frequency of these alleles between the PCB-sensitive (reference site) and -resistant (NBH) populations [51]. Here, we continue the focus on the NBH population and expand those studies to include additional populations of PCB-sensitive and –resistant fish as well as additional loci (AHR2, AHRR). The contaminated sites included two of the most well-studied DLC-tolerant populations of *F. heteroclitus*, NBH, MA, and Newark Bay, NJ (Yacht Club; YC) (Figure 1). We also included fish from two other highly contaminated sites suspected of tolerance to DLCs or other contaminants: Piles Creek, NJ (PC) [13,52], and Jamaica Bay, NY (JB) [6], as well as three reference sites: Scorton Creek, MA (SC), Flax Pond, NY (FP), and Sandy Hook, NJ (SH) (Figure 1). A companion study [53] used a ‘candidate gene scan’ approach to investigate associations between DLC tolerance and SNP markers at 59 loci in four pairs of sensitive and tolerant populations of *F. heteroclitus*, including some of the same populations examined in the present study.

We hypothesized that there would be selection in favor of AHR variants that conferred reduced sensitivity to PCBs. This might be detected as purifying selection reducing diversity at one or more of the AHR loci, or as positive selection for certain SNPs or haplotypes in all polluted sites versus reference sites. Alternatively, evidence for selection might be population-specific. By sequencing the three AHR-related loci from individuals collected at these reference and polluted sites along the Atlantic coast of North America, we compare genetic and haplotype diversity and haplotype distribution, and use multiple methods to assess signatures of positive selection at particular nucleotides. Together, our data support previous studies indicating no loss of genetic diversity in populations at polluted sites, but suggest that particular nucleotides in each gene have a signature of selection that may underlie the differences in phenotype in killifish populations.

## Results

### AHR1, AHR2, and AHRR polymorphisms

**Figure 2:**
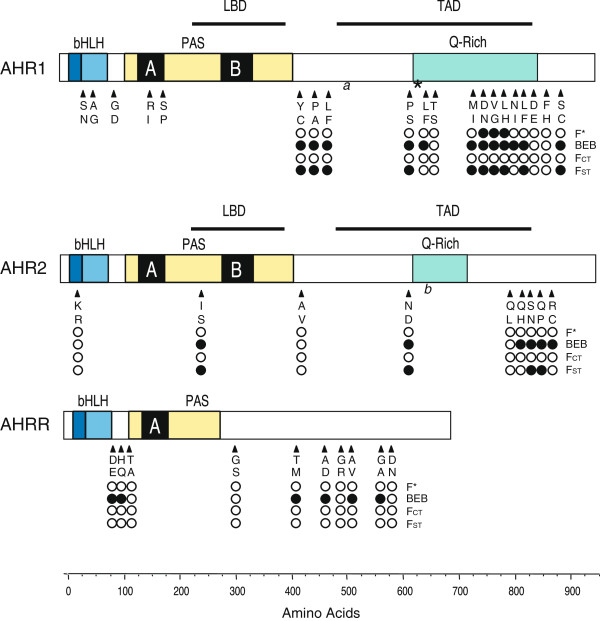
**Location of ns-SNPs in relation to AHR domain structure and results of three tests for selection at individual residues.** For each position, the two amino acids are indicated at their position in the expressed protein. A six base-pair deletion identified in AHR1 is indicated by an asterisk. Significant results from tests for selection are indicated by a black dot, while non-significant positions are indicated by a white dot. The top row (F*) indicates positions within each locus that were tested with Fu and Li’s F* statistic. The second row (BEB) indicates positively selected sites indicated by Bayesian posterior probabilities assessed in all unique haplotypes. The third row indicates sites with significantly different base frequencies in polluted versus reference populations, based on F-statistics (F_CT_). The bottom row indicates sites with significantly different base frequencies among all populations (F_ST_). In addition to the ns-SNPs in exon 10 that were used in the present analysis, the diagram for AHR1 includes ns-SNPs in the amino-terminal portion (exons 1–9) of AHR1 that were identified earlier [51] in the SC and NBH populations or in this study. The N-terminal ns-SNPs were not included in analyses for this study. The approximate locations of the AHR1_1530 SNP (*a*) and AHR2_1929 SNP (*b*) identified by Proestou et al. [53] are indicated.

*AHR1*—Previously, twenty-five single nucleotide polymorphisms (SNPs), nine of which were non-synonymous, were identified in killifish from SC and NBH [51]. Four of these nonsynonymous SNPs (ns-SNPs) occurred in the highly conserved and functionally important bHLH and PAS domains, but not at positions that are highly conserved among AHRs from different species. In the present study, full-length AHR1 cDNAs (2835 bp) were sequenced from 49 individuals from five new locations (JB, FP, PC, SH, and YC) and these data were combined with the exon 10 sequences of 52 individuals from SC and NBH determined earlier [51]. Overall, 44 SNPs, including 20 ns-SNPs, were identified. Considering only exon 10 (the longest exon: 1540 bp), there were 31 SNPs, 15 of which were non-synonymous. Twelve of these ns-SNPs were clustered in the C-terminal half of the coding sequence, surrounding the Q-rich region that is involved in transactivation (Figure 2); the resulting amino acid replacements included a mixture of conservative and non-conservative changes. In addition to the SNPs, a 6-nucleotide deletion was also identified in some fish from JB, FP, PC, SH, and YC; none of the fish from SC or NBH had the deletion. Only sequences from exon 10, which contained most of the SNPs (Figure 2), were used in subsequent population comparisons, because for some of the fish only exon 10 sequences were available.

*AHR2*—Although fish have multiple AHRs, we have observed that AHR2 is the predominant (most highly and widely expressed) form in many fishes, suggesting that AHR2 may have an important role in adaptive and toxic responses (as distinct from physiological responses) in fish [54,55]. Sequencing of 148 alleles (2860 bp each) from 74 fish at 7 locations revealed 29 SNPs, including 9 ns-SNPs. As observed for AHR1, the AHR2 ns-SNPs occurred primarily in the C-terminal half of the coding sequence, including within the Q-rich region (Figure 2); all of the C-terminal ns-SNPs resulted in non-conservative amino acid changes. Two ns-SNPs were found in the N-terminal half: a ns-SNP in the N-terminal basic region results in a conservative Lys to Arg change, and a ns-SNP in the PAS domain results in a non-conservative Ile to Ser change between the PAS-A and PAS-B repeats, in a region that is poorly conserved among species, but may have functional importance [56,57].

*AHRR*—AHRR sequences (2040 bp each) were obtained from 54 fish from the five Hudson River estuary sites. (Although AHRR is expressed in SC and NBH fish [42], no PCR products were obtained from SC or NBH fish. The reasons are not known, but could involve polymorphisms at primer target sites.) Thirty-eight SNPs, including 10 ns-SNPs, were found in the 54 sequenced fish (Table 1). The ns-SNPs occur in the C-terminal region of the coding sequence (6 ns-SNPs, 5 of which result in non-conservative aa changes), in a region after the PAS domain (1 ns-SNP), and in the region between the HLH and PAS domains (3 ns-SNPs, 2 of which result in conservative aa changes) (Figure 2).

**Table 1:** **Data summary of sequencing results for *F. heteroclitus *** **AHR1, AHR2, and AHRR from seven locations along the Atlantic coast of the United States**

	**AHR1**					**AHR2**					**AHRR**				
**Population**	**N**	**π**	**Hap**	**Hd**	**SNPs**	**N**	**π**	**Hap**	**Hd**	**SNPs**	**N**	**π**	**Hap**	**Hd**	**SNPs**
NBH	52	0.0054	10	0.836	22	38	0.0026	18	0.902	22					
SC	52	0.0057	12	0.792	24	48	0.0024	23	0.916	25					
JB	14	0.0046	14	1	18	14	0.0009	10	0.923	7	26	0.0031	10	0.812	15
FP	30	0.0043	21	0.945	23	14	0.0015	11	0.956	16	24	0.0037	18	0.971	29
PC	26	0.0055	20	0.975	23	14	0.0020	8	0.912	12	22	0.0046	21	0.966	30
SH	18	0.0058	15	0.974	24	18	0.0015	11	0.928	12	20	0.0038	17	0.984	24
YC	10	0.0026	5	0.844	11	2	0	1	0	0	16	0.0033	10	0.925	20
**Summary**	**202**	**0.0061**	**76**	**0.948**	**31**	**148**	**0.0024**	**65**	**0.970**	**29**	**108**	**0.0042**	**65**	**0.976**	**38**

Note: N = number of alleles sequenced. π = nucleotide diversity. Hap = number of haplotypes found. Hd = Haplotype diversity. SNPs = number of single nucleotide polymorphisms found. No AHRR sequences were obtained from NBH or SC fish. The “Summary” line (bold text) includes the total number of distinct haplotypes and SNPs across all populations. SNP and haplotype counts for AHR1 include sequences from exon 10 only. The unique haplotypes for each AHR locus are available in the Dryad repository, at doi:10.5061/dryad.t2888.

### AHR1, AHR2, and AHRR haplotypes and genetic diversity

**Figure 3:**
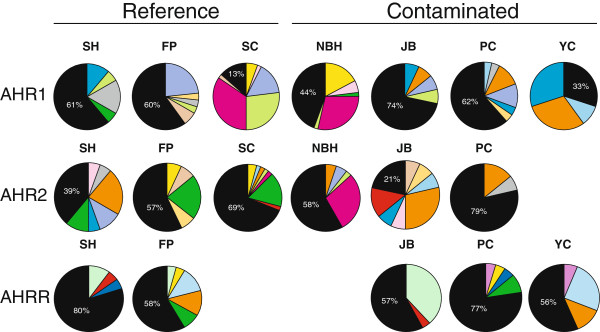
**Haplotype frequencies for each AHR locus among populations.** The black wedge indicates the percentage of site-specific haplotypes. Colored wedges indicate shared haplotypes. See Additional files 1, 2 and 3 for more detailed description of haplotypes. Overall, all populations have a higher proportion of unique haplotypes regardless of the locus or habitat type (i.e., reference or polluted).

*AHR1*—The 31 AHR1 SNPs were arranged in 76 distinct haplotypes (Table 1). Many of the haplotypes occurred only once or a few times and frequently at only one location. The percentage of site-specific haplotypes (“private haplotypes”; black wedges in Figure 3) frequently exceeded that of haplotypes shared with other populations. The highest percentage of private haplotypes was from JB, where fish contained 14 total haplotypes of which 10 were unique to this location. A few haplotypes occurred at high frequency, and at multiple sites (Figure 3; Additional file 1: Figure S1). Overall genetic diversity for exon 10 of AHR1 among the sampled fishes was high (π = 0.00613); however, there was no significant difference in nucleotide or haplotype diversity between polluted and reference locations (Table 2). The categorization of fish from JB as reference or polluted did not affect these results.

**Table 2:** **Statistical comparison of nucleotide and haplotype diversity of *F. heteroclitus *** **from polluted and reference sites**

**Character**	**Gene**	**Polluted**	**Reference**	**P-value**
Nucleotide diversity	AHR1	0.00451	0.00525	P = 0.4098
		(0.00137)	(0.000818)	
	AHR2	0.00183	0.00181	P = 0.9695
		(0.00085)	(0.00052)	
	AHRR	0.00367	0.00371	P = 0.9365
		(0.00082)	(0.000057)	
Haplotype diversity	AHR1	0.9138	0.9037	P = 0.8937
		(0.0858)	(0.0978)	
	AHR2	0.9123	0.9333	P = 0.2158
		(0.0106)	(0.0206)	
	AHRR	0.9110	0.9775	P = 0.3400
		(0.0928)	(0.00919)	

For these comparisons JB was grouped with other polluted populations. However, when JB was considered a reference site, the relationships of nucleotide and haplotypes diversity remained unchanged. Values shown are the mean and (standard deviation).

Pairwise F_ST_ comparisons of AHR1 among all populations identified approximately half (12 of 21) with significant differences between sites (Table 3). F_ST_ values varied from zero (PC vs SH) to 0.346 (YC vs NBH). There were no consistent patterns for genetic differentiation between reference and polluted sites. NBH and SC were significantly different when compared with all other populations (including the nearest other reference site FP) but not significantly different from one another.

**Table 3:** **Pairwise F**_**ST **_**values between all populations for AHR1, AHR2, and AHRR**

	**SH**	**PC**	**YC**	**JB**	**FP**	**NBH**	**SC**
***AHR1***							
SH	0						
PC	−0.00467	0					
YC	0.26461	0.1819	0				
JB	0.08137	0.04123	0.14891	0			
FP	0.08411	0.04209	**0.26796**	**0.0717**	0		
NBH	**0.2316**	**0.24953**	**0.34609**	**0.32853**	**0.30805**	0	
SC	**0.16025**	**0.15137**	**0.2387**	**0.18917**	**0.14406**	0.06992	0
***AHR2***							
SH	0						
PC	0.01223	0					
JB	0.06531	0.1143		0			
FP	**0.11681**	**0.14203**		**0.27903**	0		
NBH	**0.16712**	**0.12756**		**0.15459**	**0.21634**	0	
SC	**0.12239**	**0.12998**		**0.17283**	0.04684	**0.14197**	0
***AHRR***							
SH	0						
PC	0.09077	0					
YC	**0.13945**	0.06193	0				
JB	0.09775	**0.24847**	**0.20896**	0			
FP	**0.10319**	−0.00318	0.06668	**0.26985**	0		

Bold values indicate statistically significant differences after correcting for multiple comparisons. YC was omitted from the AHR2 analysis because of insufficient data. Similarly, AHRR sequences were not available from NBH and SC; see methods for details.

*AHR2*—The 29 AHR2 SNPs were arranged in 65 haplotypes, many occurring only once or 2–3 times at a single location. As with AHR1, haplotypes unique to single locations accounted for more than half of the total, indicating a high proportion of private alleles (Figure 3). There were also a few shared, high-frequency haplotypes (e.g., at SC and FP; SH, JB, and PC) (Figure 3; Additional file 2: Figure S2). Compared to AHR1, overall genetic diversity for AHR2 among the sampled fishes was lower (π = 0.00238). Similar to AHR1, there was no significant difference in nucleotide or haplotype diversity between polluted and reference locations overall (Table 2). The categorization of fish from JB as reference or polluted did not affect the results.

Pairwise F_ST_ comparisons among all populations revealed a similar number of significant relationships as for AHR1 (11 of 15), but with some different populations exhibiting significant genetic differentiation (Table 3). (YC could not be compared with other populations because only one individual was sequenced.) F_ST_ values varied from 0.0122 (PC vs SH) to 0.279 (JB vs FP). There were no consistent patterns for genetic differentiation between reference and polluted sites when considered as a group. However, NBH (PCB-resistant population) had significant F_ST_ values in comparison to both SC and FP (the two nearest reference populations; F_ST_ values of 0.142 and 0.216, respectively), while SC did not differ significantly from FP (F_ST_ = 0.0468).

*AHRR*—The 38 AHRR SNPs occurred in 65 haplotypes, most of which were low-frequency and site-specific (Figure 3; Additional file 3: Figure S3). Some higher-frequency AHRR haplotypes were present in most populations but these showed similar distribution among the contaminated and reference sites. Overall genetic diversity for AHRR was intermediate between AHR1 and AHR2 (π = 0.00417). There was no significant difference in nucleotide or haplotype diversity between polluted and reference locations (Table 2). As observed for AHR1 and AHR2, the categorization of fish from Jamaica Bay as reference or polluted did not affect the results.

For AHRR, pairwise F_ST_ comparisons among all populations identified half (5 of 10) that were significantly different (Table 3). F_ST_ values varied from zero (PC vs FP) to 0.270 (JB vs FP). Similar to AHR1 and AHR2, there were no consistent patterns for genetic differentiation between reference and polluted sites.

### Tests for selection

We used three methods for detecting candidate nucleotides undergoing selection: Tajima’s D and Fu and Li’s F*, likelihood ratio tests, and position-specific F-statistics. Tajima’s D was not significant for any of the loci (AHR1: D = 1.298, p > 0.1; AHR2: D = 0.841, p > 0.1; AHRR: D = 0.848, p > 0.1). However, Fu and Li’s F* test was significant for AHR1 (F = 2.069, p < 0.02) but not for AHR2 (F = 1.024, p > 0.1) or AHRR (F = 0.319, p > 0.1). An F-statistic significantly greater than zero for AHR1 indicated an excess of intermediate frequency alleles. Using the sliding window approach for AHR1, three nucleotide positions, clustered in the Q-rich region of the transactivation domain (TAD), were significant (top row of AHR1 in Figure 2).

The likelihood ratio tests implemented with codeml in the PAML software suite provided evidence for positive selection shaping the frequency of nonsynonymous polymorphisms in all three loci. Both tests (M1a vs. M2a; M7 vs. M8) were highly significant (p < 0.001) for each locus. For both comparisons, the Bayes Empirical Bayes (BEB) method identified the same set of nucleotides as under positive selection; these included 12 residues in AHR1, 6 in AHR2, and 6 in AHRR (second row in Figure 2). Each of these residues was inferred with high probability (p > 0.99) to be under strong selection, with ratios of nonsynonymous substitutions to synonymous substitution (ω) greater than 9. The 12 residues identified in AHR1 represented 80% of the ns-SNPs sequenced from these populations and 2.2% of the total residues. These sites are dispersed throughout the sequenced region of exon 10 with intervening sites showing no evidence of positive selection. For AHR2, one identified residue was in the region between the two PAS domains and the remaining residues were in the TAD or further in the C-terminus; four of these were successive substitutions in the C-terminus. The six positively selected sites for AHRR were scattered throughout this locus, representing 60% of ns-SNPs, and included two residues in the region between the bHLH and PAS domains. Over all three loci, 1.1% of codons and more than 70% of nonsynonymous substitutions were identified as being under positive selection.

Locus-by-locus AMOVA was used to test for significant differences in SNP frequencies between populations classified as polluted versus reference (F_CT_, third row of Figure 2). These tests did not identify any ns-SNPs in AHR1 with significant differences in these two habitat types. These results were not affected by classification of JB as a polluted or reference site. However, nucleotide frequencies were significantly different among populations for 10 of 15 ns-SNPs when all geographic locations were included (F_ST_; fourth row of Figure 2) or for 9 sites within reference or polluted (F_SC_) (Additional file 4: Table S1). Synonymous substitutions, like the ns-SNPs, did not show significant variation between reference and polluted populations (Additional file 4: Table S1, F_CT_ column), but they did show some significant F_ST_ and F_SC_ values.

As observed for AHR1, F-statistics for AHR2 and AHRR identified no ns-SNPs as having significant differences in frequencies between populations classified as polluted versus reference. Four AHR2 ns-SNPs had significant F_ST_ values (Figure 2) and a single ns-SNP located in the PAS domain region of AHR2 (an I/S replacement) had a significant F_SC_ value (Additional file 4: Table S1). For AHRR, no ns-SNPs had significant F_SC_ or F_ST_ values. Synonymous substitutions in both AHR2 and AHRR were also not significant when populations were grouped as reference or polluted, but two synonymous SNPs showed significant F_SC_ and six showed significant F_ST_ (Additional file 4: Table S1).

Comparing results of these different tests for ns-SNPs under selection, three ns-SNPs in the Q-rich domain of AHR1 were identified by three tests (Figure 2). An additional seven ns-SNPs in AHR1 and four ns-SNPs in AHR2 were identified by two tests. No ns-SNPs in AHRR were identified by more than one test.

**Figure 4:**
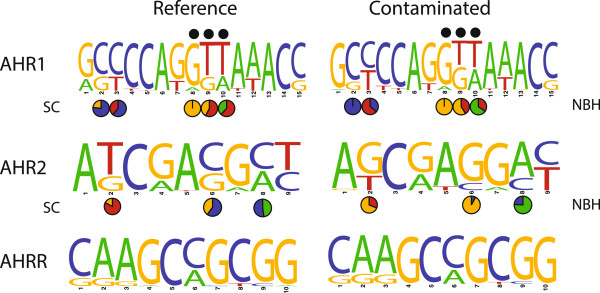
**Base frequencies for all identified ns-SNPs in the three AHR loci.** Black dots above the frequency plots indicate ns-SNPs identified by more than one test for selected residues (see Figure 2). Sequence logos indicate the average base frequencies in populations classified as reference (left) and contaminated (right). Pie charts indicate the base frequencies in two focal populations in Massachusetts (Scorton Creek, a clean site, and New Bedford Harbor, a polluted site) for ns-SNPs identified in multiple selection tests as well as for two additional ns-SNPs in AHR1 (positions 2 and 3) and three in AHR2 (positions 2, 6, and 8). These later ns-SNPs were not identified in the multiple tests for selection but comparisons between these two sites showed strong divergence in base frequency between the SC and NBH populations.

We compared the base composition between reference and contaminated sites for each of the ns-SNPs in all three loci (Figure 4). For the three ns-SNPs identified by three tests for selection (black dots above the nucleotide position), we compared the frequency of each nucleotide in the SC and NBH populations. Despite overall similarity in base frequency between reference and polluted sites, the base frequencies varied considerably between SC and NBH for two of these ns-SNPs in AHR1: ns-SNPs 9 and 10. Two other positions (ns-SNPs 2 and 3) also show base frequency differences between SC and NBH and in the comparisons of all populations were identified as under selection by both likelihood ratio tests and AMOVA-F_ST_. Three positions in AHR2 showed dramatic differences in base composition between SC and NBH fish: ns-SNP 2 (NBH: 31.5% T, SC: 80% T), ns-SNP 6 (NBH: 8% C; SC: 60% C), and ns-SNP 8 (NBH: 76% A; SC: 48% A) (Figure 4). Two of these (ns-SNPs 2 and 8) were also identified by both likelihood ratio tests and AMOVA-F_ST_ as under selection in the comparisons of all populations.

### Isolation by distance

**Figure 5:**
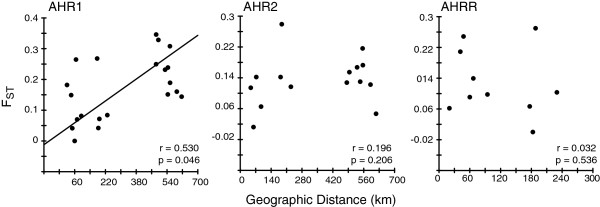
**Mantel tests of isolation-by-distance.** The regression for AHR1 was the only significant relationships between geographic distance and genetic similarity. The other comparisons (AHR2 and AHRR) were not significant.

The sampled populations in this analysis spanned a total geographic distance of more than 600 km along the Atlantic coastline of the United States (see Additional file 5: Table S2 for geographic distances). Statistical tests for isolation-by-distance resulted in a significant regression when AHR1 diversity was compared among all sampled fishes (r = 0.530, p = 0.046), a relationship consistent with a neutral expectation, although distance explains only part of the difference in genetic diversity. The regressions for AHR1 remained positive when fish were separated by type of habitat (i.e., reference vs. polluted); however these statistical tests were not significant (data not shown). In contrast to the results for AHR1, results for AHR2 and AHRR indicated non-significant relationships (possible non-neutral evolution) both when comparing among all estuaries (Figure 5) and when sites were separated by habitat type (data not shown). An alternative possibility, perhaps likely for AHRR, is that sample sizes were too small to detect isolation by distance.

## Discussion

The repeated evolution of resistance to DLCs in widely separated populations of *F. heteroclitus* along the U.S. east coast provides an opportunity to understand the mechanistic basis for rapid adaptation to anthropogenic environmental change. There is strong evidence—initially from the widespread loss of inducibility of AHR-regulated CYP1A [reviewed in 6] and subsequently confirmed by gene expression profiling [26,58,59]—that this adaptation involves altered sensitivity of the AHR-dependent signaling pathway. Thus, we used a candidate gene approach and focused on three known AHR-related genes in seven populations of *F. heteroclitus*. Our analysis of the sequence data from all seven locations reveals a complex pattern of selection at the three loci. Because our primary focus has been on the NBH Superfund site [23,42,51,59-63], we also examined the patterns of variation at NBH and its two nearest reference sites, SC and FP.

### Comparisons of seven populations from polluted and reference sites

Three AHR-related loci (AHR1, AHR2, AHRR) from *F. heteroclitus* inhabiting seven estuaries along the U.S. east coast contain a large number of polymorphisms, many of which result in changes in the encoded amino acids. Overall, 1.5% of the nucleotide positions were variable among the sequences analyzed in this study, and 38% of the SNPs were nonsynonymous. In contrast, AHR2 in tomcod sampled from three sites (60 alleles total) showed very low nucleotide variability (0.1%) [64]. For comparison, 3.5% of the nucleotide positions were variable in AHR coding sequences from 13 inbred strains of mice (*Mus musculus*) [65]. By contrast, the human AHR (0.4%) exhibits much less variability than either mouse or killifish AHRs [66,67].

In previous studies examining inter-specific and intra-specific variability in AHR sequences [45,65,68], the most highly conserved region is the basic-helix-loop-helix (bHLH) domain, which is involved in DNA binding and protein dimerization [69,70]. The Per-Arnt-Sim (PAS) domains, required for ligand-binding and protein-protein interactions [69,70], are also well conserved, whereas the C-terminal half of the protein that harbors transactivation domains [71,72] is more variable [45,65,68]. Our results showing that the majority of ns-SNPs are in the C-terminal half of the sequences or between bHLH and PAS domains (Figure 2) are consistent with these earlier results.

The genetic diversity of these *F. heteroclitus* populations at these three loci is strongly partitioned among locations, but there are no significant differences in nucleotide diversity between populations inhabiting polluted habitats versus those at relatively clean habitats. Similarly, each locus is represented by dozens of haplotypes that exhibit a high degree of location-specific distribution but, again, with no consistent differences in haplotype diversity in polluted versus reference habitats. In previous studies, examination of other sequence-based markers, microsatellites, and anonymous markers has led to the conclusion that there is restricted gene flow among these populations (i.e., genetic structure) and that populations inhabiting pollutant-impacted sites show no strong signature for a genetic bottleneck (i.e., loss of genetic diversity) [29,32,33,53,73]. Our results show that these conclusions also pertain to the three AHR-related gene loci. Thus, the populations exhibit strong genetic structure at these loci but no loss of nucleotide or haplotype diversity in populations classified as “polluted.”

Despite an overall similarity in genetic diversity between reference and polluted populations, a suite of tests suggested that some loci and certain polymorphisms may be under selection. The three statistical methods we used test for deviations from neutrality using different metrics. Tajima’s D and Fu and Li’s F* statistics test for a statistically significant excess or reduction of allele frequencies among sampled individuals. The likelihood/BEB method tests for an excess of nonsynonymous substitutions compared to synonymous substitutions along particular haplotype lineages, at each position. The comparative F-statistics test for significant differences in base-frequencies among populations (F_ST_) or between groups of populations classified as reference or polluted (F_CT_). These tests may identify different sets of nucleotides potentially under selection. However, when the same nucleotide is identified by more than one test, it increases confidence that it has been shaped by selection. The most consistently identified three SNPs, identified by three tests, were located in the Q-rich region of AHR1, in a region of exon 10 associated with the transcriptional activation function [71,72]. Other ns-SNPs in exon 10 of AHR1 and in AHR2 were identified by two tests (Figure 2).

The position-specific likelihood ratio tests identified more residues under selection when compared with the other tests. Generally, ratio comparisons of nonsynonymous and synonymous SNPs are considered conservative tests for positive selection [74]. Additionally, our results were inferred through analysis of haplotypes in a phylogenetic framework, thus allowing a more accurate representation of the evolution of particular nucleotides within the lineage of an allele. Whether all identified SNPs represent true positives is uncertain, in part due to the limitations of our data set. One limitation is that recombination within loci can hamper interpretation of residues under selection in tree-based analyses by increasing the proportion of false positives [75]. An initial test for recombination at each of the three loci using the GARD test [76] suggests potential recombination events in the sampled individuals from this study. These analyses indicate that one (AHR1, AHRR) or two (AHR2) recombination events likely have occurred in our sampled sequences. Such a low frequency of recombination is unlikely to cause false positives in nucleotide-specific tests for selection [75]. Likelihood ratio tests are also sensitive to data sets in which polymorphic sites are not independent, and because of linkage the BEB analysis may over-represent residues under selection [77]. Thus, some SNPs identified as undergoing selection may represent SNPs in linkage disequilibrium with neighboring nucleotides for which selection was operating. Discerning among these possibilities would be assisted by sequencing additional loci from these individuals.

More broadly, our results provide a mixed assessment of which AHR locus may represent the best candidate for explaining evolved resistance in natural populations of *F. heteroclitus*. Similar to population genetics studies of other *F. heteroclitus* loci [32,53,73,78-80], at each AHR locus we observed a high number of polymorphisms that segregate among populations, with many haplotypes restricted to individual locations. Such a large proportion of geographically restricted genetic diversity reflects this species’ large population sizes and relatively limited migration between adjacent locations [10]. AHR1 and AHR2 are possible candidates for explaining the mechanism of molecular adaptation by populations to polluted environments. For the statistical tests for selection, AHR1 had proportionally larger numbers of ns-SNPs with evidence of selection. However, AHR1 diversity showed a significant (though weak) relationship with geographic distance, a result consistent with either neutral evolution (isolation by distance) or selection pressure that correlates with latitude (e.g., temperature, photoperiod) [81]. However, DLC contamination is not correlated with latitude, i.e., the polluted sites in this portion of *F. heteroclitus*’ range, as well as along the Atlantic coast, are interspersed among clean sites. Thus, the Mantel test result for AHR2, showing the lack of a relationship of genetic diversity with geographic distance, is more consistent with adaptation to local environments. On the other hand, we found fewer AHR2 ns-SNPs to be under selection, although some of these variable positions showed significant differences when comparing all populations (F_ST_) and in comparison of NBH and SC (see below). Experimental tests to empirically determine functional characteristics (e.g., PAH binding, protein-protein interactions) of the diverse AHR1 and AHR2 allelic variants would help to discern the role of SNPs in adaptation in these populations and to develop hypotheses about the role of particular haplotypes in polluted and reference populations of *F. heteroclitus*.

### NBH *versus* reference population comparisons

Examination of AHR diversity in multiple populations, including several exhibiting resistance to DLCs [6], revealed evidence for AHR loci and specific SNPs under selection, but the population genetic data are complex and their interpretation is not straightforward. A limitation of this multi-population approach is that resistance is likely to have evolved independently in the different resistant populations and may involve different loci or different SNPs or haplotypes under selection. In addition, our classification of locations as “polluted” combined locations with very different types of pollution (PCBs, dioxins, PAHs, metals), and included a population (PC) for which DLC resistance has not yet been assessed. It is useful, then, to also take a more focused look at the population of greatest interest in our studies, the one inhabiting the NBH Superfund site, and the two nearest reference populations, SC and FP, thus minimizing effects of geographic distance on the genetic data.

Consistent with the Mantel test showing isolation-by-distance for AHR1 across all populations, the NBH and SC populations did not show strong genetic differentiation at this locus, but each had significant pairwise F_ST_ values in comparison to all of the more distant populations (including FP). By contrast, for the AHR2 locus NBH had significant F_ST_ values in pairwise comparisons to SC and FP (Table 3), while the two reference sites did not differ significantly from each other, despite the fact that they are farther apart from each other than either is from NBH. These results were supported by the distinct pattern of haplotype frequencies at NBH as compared with SC or FP (Figure 3) and by the identification of three AHR2 ns-SNPs for which NBH and SC differ substantially (Figure 4). Thus, examination of these three populations points to specific AHR2 SNPs and haplotypes as being associated with the PCB-resistant phenotype.

One interesting result is that, for both AHR1 and AHR2, neither specific haplotypes nor the SNPs exhibiting evidence for selection were fixed in DLC-resistant fish populations, raising questions about their contribution to the resistant phenotype. One possibility is that these loci individually have relatively small effect and are part of a larger polygenic adaptation response [82]. Alternatively, there could be multiple haplotypes at one of these loci (e.g., AHR2) that confer resistance. Such a situation could arise from selection on pre-existing (standing) genetic variation, in which one or more SNPs conferring reduced AHR function exists in multiple haplotypes in the population prior to environmental change, and selection leads to fixation of multiple alleles (soft sweep [83-85]). Population genomic studies will help to distinguish between these possibilities.

### Role of AHR2 in controlling susceptibility of fish to DLC effects

Fish have multiple AHR genes, classified in two clades, AHR1 and AHR2 [86]. The functions of AHR1 and AHR2 are not completely understood, but AHR2 is the most likely candidate for a resistance locus, based on several lines of evidence. First, studies using gene-specific knock-down in zebrafish embryos have shown that AHR2 controls the induction of CYP1A and sensitivity to developmental toxicity of TCDD, PCBs, and PAHs in this species [37,38,87]. Second, AHR2 was one of the candidate genes emerging from a genome-wide QTL screen for genes controlling PCB cardiotoxicity in zebrafish embryos [88]. Third, and more directly relevant to the species of interest in the current study, knock-down of AHR2 in embryos of *F. heteroclitus* provided partial protection against the teratogenic effects of PAHs and PCBs [89].

In addition to the experimental studies cited above, two recent population-level studies suggest AHR2 as a resistance locus. In an independent analysis being published as a companion paper in this journal [53], a ‘candidate gene scan’ investigation of associations between DLC resistance and SNP markers at 59 loci in four pairs of sensitive and tolerant populations of *F. heteroclitus* identified AHR2 as one of two loci under selection (the other was CYP1A) [53]. There is partial overlap in the populations studied by Proestou et al. [53] and in the present paper (NBH, FP, SH, YC/NWK) but the other populations examined were specific to each study (us: SC, JB, PC; Proestou et al.: BI, BP, ER, KC). In Proestou et al. [53], SNPs in both AHR1 (AHR1_1530) and AHR2 (AHR2_1929) exhibited evidence of selection (significant F_ST_ values) in 3 of 4 population pairs, including NBH and its reference site. In our study, AHR1_1530 also had a significant F_ST_ value in a locus-by-locus AMOVA and it is located just downstream from three ns-SNPs also exhibiting evidence for selection (Figure 2; Additional file 4: Table S1). Although the AHR2_1929 SNP did not have a significant F_ST_ value in our study (Additional file 4: Table S1), it was near a ns-SNP that did (AHR2_1813; N/D amino acids in Figure 2). Additional evidence for selection at the AHR2_1929 SNP in Proestou et al. [53] came from patterns of minor allele frequencies between pairs of populations and the identification of this SNP as the only outlier after F_ST_ modeling of pooled sensitive and tolerant populations [53].

A second study, in another fish species showing population-specific evolution of PCB resistance, also implicated the AHR2 locus. Atlantic tomcod (*Microgadus tomcod*) inhabiting the PCB-contaminated Hudson River were nearly monomorphic for an AHR2 variant with reduced capacity to bind and be activated by halogenated AHR ligands such as TCDD or PCB-126 [64]. The AHR2 variant in Hudson River fish was characterized by a 2-amino acid deletion, just downstream from the PAS domain, that was proposed to alter the ligand-binding affinity or stability of the AHR2 protein in these fish. A similar deletion was not found in the AHR2 variants of NBH killifish in our study, but a SNP within the PAS domain and several near the C-terminal transactivation domain emerged as potentially under selection and with distinct patterns in NBH fish as compared to the reference sites (Figures 2, 4).

Based on our results and those described above [53,64,88,89], we suggest that evolution of resistance to PCBs in fish may converge on a common target gene, AHR2, but that the specific molecular changes may differ between species, and perhaps also within a species among populations that have independently evolved the resistant phenotype (for other examples, see [90,91]). Nevertheless, changes in other loci—including paralogous AHR loci (see below) as well as other loci encoding proteins involved in the mechanism of dioxin toxicity—may also play a role in conferring the resistant phenotype. Population genomic surveys currently underway will help illuminate such possibilities.

Since completion of this work, through transcriptome sequencing [59], we have identified two additional AHR loci in *F. heteroclitus*, paralogs of the AHR loci studied here. (The differences between paralog sequences are sufficiently large so that the paralogs could not have interfered with the sequencing or SNP determinations reported in this paper.) Multiple AHRs, often occurring as pairs of paralogous AHR1 and/or AHR2 forms, have been identified in other species of fish including *Danio rerio* (zebrafish), *Takifugu rubripes* and *Tetraodon nigroviridis* (pufferfishes), *Oryzias latipes* (medaka), and salmonids (reviewed in [86]). Consistent with phylogenetic relationships (unpublished analysis) and the nomenclature we have used for other fish AHRs [86], the original killifish AHR genes (the focus of this paper) have been designated AHR1a and AHR2a; the novel AHR genes are AHR1b and AHR2b. The function and expression patterns of these new AHRs are not known, but are under active investigation in our laboratory. Sequencing and assembly of the *F. heteroclitus* genome has revealed that AHR1a and AHR2a occur in tandem (~14 kb apart), as do AHR1b and AHR2b (~4 kb apart), as we have described for other fish AHR1/AHR2 pairs [86,92]. Linkage of AHR1a and AHR2a may have influenced the patterns of diversity and evidence for selection obtained in our study and that of Proestou et al. [53], for example by causing both AHR1 and AHR2 to display evidence for selection even if only one of these genes may be involved in the mechanism of resistance. Clearly, additional research will be needed to determine the function of the new AHRs and the possible role of all four AHR genes in evolved resistance to PCBs and related chemicals.

## Conclusion

The data presented here suggest that *F. heteroclitus* populations in reference and polluted sites have similar genetic diversity, with no evidence for genetic bottlenecks in populations inhabiting polluted locations. However, the populations exhibit strong genetic structure at all three AHR-related loci, and for AHR2 the NBH population exhibits significant genetic differentiation from its two nearby reference sites. In addition, the data revealed positive selection at specific nucleotides in AHR1 and AHR2, and specific AHR2 SNPs and haplotypes that are associated with the PCB-resistant phenotype in the NBH population. The results suggest that AHRs, and especially AHR2, may be recurring targets for selection during local adaptation of fish to dioxin-like aromatic hydrocarbon contaminants, although the specific molecular changes may vary among independently adapting populations or species.

## Methods

### Site selection, fish collection, and sample processing

*F. heteroclitus* (26 fish per site) were collected from New Bedford Harbor, MA, USA (NBH; PCB-contaminated site) and Scorton Creek, Sandwich, MA, USA (SC; reference site for NBH) in May-June, 2003 as part of a previous study on AHR1 alleles [51]. Additional *F. heteroclitus* (15 fish per site) were collected between June and October 2002 from five sites within or near the lower Hudson River ecosystem (Figure 1). The polluted sites were: Newark Bay, NJ [Roanoke Yacht Club (YC) [14,15]], Piles Creek, NJ (PC) [13,52], and Jamaica Bay, NY (JB) [6] (Figure 1). The additional reference sites were: Sandy Hook, NJ (SH) [6] and Flax Pond, NY (FP) [6,15]. These sites were chosen because the PCB sensitivities of most of their *F. heteroclitus* populations have been characterized and sediment PCB levels have been measured [6,22-24,32], allowing us to classify them as polluted or reference. The exception was Piles Creek, a highly contaminated site with killifish that have evolved resistance to methyl mercury [13,52] but also show some abnormalities [93,94]; DLC resistance has not yet been assessed for this population. Fish were collected and tissues sampled using protocols approved by the Woods Hole Oceanographic Institution’s Animal Care and Use Committee (Animal Welfare Assurance Number A3630-01).

Note on number of alleles analyzed: A formal power analysis was not performed prior to conducting these studies. Although the number of alleles sampled was sufficient to detect selection despite the high genetic diversity, sampling of a greater number of alleles from each site may have allowed us to identify additional SNPs potentially under selection. The number of alleles sampled here (10–52 per population) is in line with numbers used in other studies seeking evidence for adaptive genetic change, for example in color patterns in beach mice (8–40 alleles per population [95]), tomcod exhibiting resistance to PCBs (20–124 alleles per population [64]), and rats evolving resistance to warfarin (variable number of alleles per population [96]).

### Oligonucleotide primers

Primers were synthesized by Midland Certified Reagent Company, Inc. (Midland, Texas), Life Technologies, Inc. (Rockville, MD) or Integrated DNA Technologies, Inc. (Coralville, IA). Primer sequences are listed in Additional file 6: Table S3.

### RNA isolation, RT-PCR, and DNA sequencing

Total RNA was isolated from combined soft tissue of individual fish using RNA STAT-60 (Tel-Test B, Inc.; Friendswood, TX). RNA quality was assessed by gel electrophoresis. PolyA^+^ RNA was purified with the MicroPoly (A) Purist kit (Ambion, Grand Island, NY). First-strand cDNA was synthesized from 2 μg of polyA^+^ RNA using the Omniscript Reverse Transcription kit (Qiagen, Valencia, CA). When possible, we amplified the full coding sequences using a single pair of primers (Additional file 6: Table S3); in some cases, we used two pairs of oligonucleotide primers to produce overlapping fragments of ~1500 bp each. For these PCR reactions, 1 μl of undiluted cDNA was used with the amplification primers indicated in Additional file 6: Table S3, using Advantage 2 polymerase mix (Clontech, Mountain View, CA). PCR conditions were: For AHR1: 95°C, 1 min.; 5 cycles of [95°C, 5 sec., 73°C, 5(1.5*) min.], 5 cycles of [95°C, 5 sec., 71°C, 5(1.5*) min.], 35 cycles of [95°C, 5 sec., 69°C, 5(1.5*) min.], 72°C, 7 min., For AHR2: 95°C, 1 min., 5 cycles of [95°C, 5 sec., 72°C, 3(2.5*) min]; 5 cycles of [95°C, 5 sec. 70°C, 3(2.5*) min.]; 40(35*) cycles of [95°C, 5 sec., 68°C, 3(2.5*) min.]; 72°C, 7 min. For AHRR: 95°C, 1 min.; 5 cycles of [95°C, 5 sec., 72°C, 2.5 min]; 5 cycles of [95°C, 5 sec. 70°C, 2.5 min.]; 35 cycles of [95°C, 5 sec., 68°C, 2.5 min.]; 72°C, 7 min (* indicates program used for amplification from New Bedford Harbor and Scorton Creek samples). PCR products were initially confirmed by gel electrophoresis, and then purified with the MinElute PCR Purification Kit (Qiagen). After purification, PCR products were sequenced directly on an ABI 3730 capillary sequencer (Marine Biological Laboratory, Woods Hole, MA) using gene specific oligonucleotide primers (Additional file 6: Table S3). For unknown reasons, sequencing was not successful for all individuals from each site. This was particularly true for AHR2 sequences from YC fish, and AHRR sequences from SC and NBH fish.

### Sequence analysis

Sequences were initially scanned with Editview 1.0.1 and imported into Sequencher 4.1, which aligns the sequences and allows for the direct comparison of each electrophoretogram. The nucleotide and codon number were noted for each polymorphic site, and whether the base change resulted in a change in the amino acid at that site (non-synonymous SNPs).

### Haplotype reconstruction and data analysis

Haplotypes were inferred using PHASE v.2.02 [97,98], which implements a Bayesian statistical method to reconstruct haplotypes from unphased genotype data. The Bayesian approach used in PHASE is more accurate than the widely used Expectation-Maximization (EM) algorithm and other methods [98,99]. The output includes a summary of results with an estimate of population haplotype frequency and lists of the most probable haplotype pairs for each individual. Several PHASE runs (4 or 5) using different values for the seed of the random number generator (−S function) were performed for each gene. The number of iterations, thinning interval, and burn-in values were increased to 1000, 10, and 1000, respectively, from the default values. The results from the multiple runs were compared with respect to the allele frequencies to check for consistency. Also, the goodness of fit outputs from different runs were compared by single-factor ANOVA to assess variation among runs.

TCS software [100] was used to estimate the genealogical relationships among the haplotypes. TCS uses the method of Templeton *et al.*[101] to reconstruct phylogenies while taking into account recombination events. Haplotype frequency data were incorporated into the TCS output.

### Genetic diversity and data analysis

Genetic diversity of sampled populations was assessed by comparing nucleotide and haplotype diversity within and between populations. Both measures of diversity were calculated with DnaSP v.5 [102]. Genetic diversity measures were statistically compared with t-tests (JMP) by categorizing fish populations into two types: clean (reference; SC, FP, SH) and polluted (NBH, JB, PC, YC). While JB killifish have been shown to be sensitive to DLCs [6], site contamination and the presence of some PCB “hot spots” confounds the categorization as a reference site. Thus, alternate statistical comparisons were performed with this population categorized as either polluted or reference. To test for population genetic structure, pairwise measures of genetic differentiation among populations were calculated with F-statistics for each gene (Arlequin v.3 [103]). Significant relationships were assessed at p = 0.002 for AHR1 and AHR2 and p = 0.005 for AHRR to account for multiple comparisons (p = 0.05/number of comparisons).

### Tests for selection

Three tests using all SNPs (synonymous and nonsynonymous) were conducted to assess potential signatures of selection among these three loci. First, summary-statistic based methods (i.e., Tajima’s D, Fu and Li’s F*) were analyzed in DnaSP. For these analyses, full length, aligned sequences for AHR2 and AHRR and exon 10 of AHR1 were used as input. [For NBH and SC fish, only exon 10 sequences were available for AHR1; exon 10 contains the majority of the SNPs at this locus [51].] The two methods differ in that Fu and Li’s F* is based on the difference between the number of singleton polymorphisms and the number expected under neutrality, given the number of segregating positions, while Tajima’s D takes into account the difference between average pairwise diversity between sequences. Fu and Li’s F* test can therefore account for some degree of population structure [104], which is expected for this species. In either test, a value that significantly exceeds zero indicates an excess of intermediate-frequency alleles that could result from balancing selection, while negative values indicate an excess of low frequency alleles, which may indicate purifying selection. For each test, we used the sliding window (100 bp window, 25 bp step) implemented in DnaSP to investigate whether particular regions of each gene showed significant signatures for differentiation among the sampled populations. Significance was assessed at p < 0.05.

Second, tree-based methods to test for positive selection were implemented in PAML v.4 (codeml, [105]). For these analyses, all unique haplotype sequences for each locus were used. For these position-specific tests for selection, a phylogenetic tree was required. For each locus, best trees for AHR1, AHR2, and AHRR were produced for all unique haplotypes with maximum likelihood analyses (RAxML, [106]) using the best model for nucleotide substitutions (jModelTest [107]). Support for nodes was determined with 1000 bootstrap replicates. Each analysis resulted in a single best tree with low bootstrap (< 50) for most nodes. Two pairs of likelihood ratio tests [105][108] were used to test for evidence of positive selection. In the first pair we compared the null model of nearly neutral evolution (M1a) to the alternate model of positive selection (M2a). The second test compares a model of a beta-distributed variable selection pressure (M7) to the alternate, which includes positive selection (M8). Codons under selection were determined with posterior probabilities determined by the Bayes Empirical Bayes (BEB) method [108]. For tests of each locus we performed the repeated comparisons with different codon frequency models to see if the results were influenced by this parameter. The results from these sensitivity tests found that this parameter did not change the nucleotide positions inferred to be under selection.

Third, we used analysis of molecular variance (AMOVA) to determine differentiation among populations and between sets of populations classified as polluted or reference. All nucleotide variants in our analyses are from the coding region of each transcript and represent a combination of synonymous and nonsynonymous polymorphisms, which may display variable signals of population structure and be under different degrees of selection. Selection may result in significant differences in F-statistics for particular nucleotides if they are not evolving under neutral conditions. The locus-by-locus AMOVA feature of Arlequin [103] was used to determine F-statistics for each variable position. We were especially interested in nucleotide positions that were significantly different between the set of populations classified as polluted as compared to those classified as reference (F_CT_) but also calculated variation among all populations (F_ST_) and among populations within each class (F_SC_). To reduce Type 1 errors, we assessed significant differences at p < 0.0001.

We compared the frequency of polymorphisms identified in at least one test for selection in fish collected from New Bedford Harbor and Scorton Creek, Massachusetts. For these positions, we constructed sequence logos (Weblogo v. 3 [109]) to display the frequency of each base from all fishes categorized by reference or polluted population. We then calculated the frequency of these bases in the two focal populations at these positions as well as other positions at which there were large differences in base frequency.

### Isolation by distance

Statistical tests of isolation-by-distance were carried out for each gene among all populations and by studying populations from reference and polluted sites separately. Geographic distances between populations were determined by calculating a smoothened coastal distance between locations that ignored small inlets, when present. Pairwise genetic distances (multilocus F_ST_) were calculated between each population pair with Arlequin. Geographic and genetic distances were regressed with a web-implementation of Isolation By Distance v3.16 [110] with 1000 randomizations. Regressions were additionally completed using the linearized value of F_ST_/(1-F_ST_) in place of F_ST_. The relationships were unchanged and we only report results using F_ST_ for genetic distance.

### Availability of supporting data

The data supporting the results of this article (unique haplotypes for each AHR locus) are available in the Dryad Digital Repository: http://dx.doi.org/10.5061/dryad.t2888.

## Abbreviations

TCDD: 2,3,7,8-tetrachlorodibenzo-p-dioxin; PCB-126: 3,3′,4,4′,5-pentachlorobiphenyl; ARNT: AHR nuclear translocator; AHRR: AHR repressor; AMOVA: Analysis of molecular variance; AHR: Aryl hydrocarbon receptor; BEB: Bayes Empirical Bayes; CYP1A: Cytochrome P450 1A; DLC: Dioxin-like compound; FP: Flax Pond; JB: Jamaica Bay; NBH: New Bedford Harbor; YC: Newark Yacht Club; ns-SNPs: Nonsynonymous SNPs; PC: Piles Creek; PCB: Polychlorinated biphenyl; PCDD: Polychlorinated dibenzo-*p*-dioxin; PAHs: Polycyclic aromatic hydrocarbon; SH: Sandy Hook; SC: Scorton Creek; SNPs: Single nucleotide polymorphisms; TAD: Transactivation domain.

## Competing interests

The authors declare that they have no competing interests.

## Authors’ contributions

AMR carried out the population genetic and statistical analyses and drafted the manuscript. SIK participated in the design of the study, sequencing and sequence analysis, carried out the haplotype analyses, and edited the manuscript. DGF participated in sequencing and sequence analysis and edited the manuscript. BRE participated in the design of the study, collection of fish, and sequencing. DN participated in the design of the study, led the collection of fish, and edited the manuscript. DC participated in the collection of fish. VMV participated in the data analysis involving geographic distances. MEH conceived of the study, participated in its design and coordination, helped to draft the manuscript, and edited the manuscript. All authors read and approved the final manuscript.

## Supplementary Material

Additional file 1: Figure S1 AHR1 haplotype frequencies and distributions. Haplotypes were reconstructed using PHASE and genealogical relationships among the haplotypes were estimated using TCS software, as described in *Materials and Methods*. In the top panel, circles refer to unique haplotypes, wedges are colored by sampled population, and numbers within wedges refer to the number of alleles with that haplotype in the population represented by that color. Numbers outside of the circles refer to the haplotype number as shown in the bottom panel. In the bottom panel, black wedges indicate the percentage of site-specific (SS) haplotypes at each site and colored wedges indicate haplotypes shared among populations. Click here for file

Additional file 2: Figure S2 AHR2 haplotype frequencies and distributions. Haplotypes were reconstructed using PHASE and genealogical relationships among the haplotypes were estimated using TCS software, as described in *Materials and Methods*. For additional description, see legend to Additional file 1: Figure S1. Click here for file

Additional file 3: Figure S3 AHRR haplotype frequencies and distributions. Haplotypes were reconstructed using PHASE and genealogical relationships among the haplotypes were estimated using TCS software, as described in *Materials and Methods*. For additional description, see legend to Additional file 1: Figure S1. Click here for file

Additional file 4: Table S1 Locus-by-locus F-statistics for AHR-related loci from *Fundulus heteroclitus*. The locus-by-locus AMOVA feature of Arlequin was used to determine F-statistics for each variable nucleotide. Nucleotide positions that were significantly different among populations within each class (F_SC_), among all populations (F_ST_), and between the set of populations classified as polluted as compared to those classified as reference (F_CT_) were assessed using a significant cutoff of p < 0.0001. SS=synonymous site; NS=nonsynonymous site; nonsynonymous positions are also shaded. Nucleotide number (nt#) refers to the position with respect to the ATG translational start (A=1) unless otherwise specified. Click here for file

Additional file 5: Table S2 Pairwise geographic distances (km) for locations where *Fundulus heteroclitus* were collected for this study. Click here for file

Additional file 6: Table S3 PCR primers. Click here for file
