# Supplementary figures and images for: Genetic variation at aryl hydrocarbon receptor (AHR) loci in populations of Atlantic killifish (Fundulus heteroclitus) inhabiting polluted and reference habitats
Source: BMC Evol Biol. 2014 Jan 14;14:6. doi: 10.1186/1471-2148-14-6 (PMC3899389; doi:10.1186/1471-2148-14-6)

# AHR1 SNP Haplotype Distribution

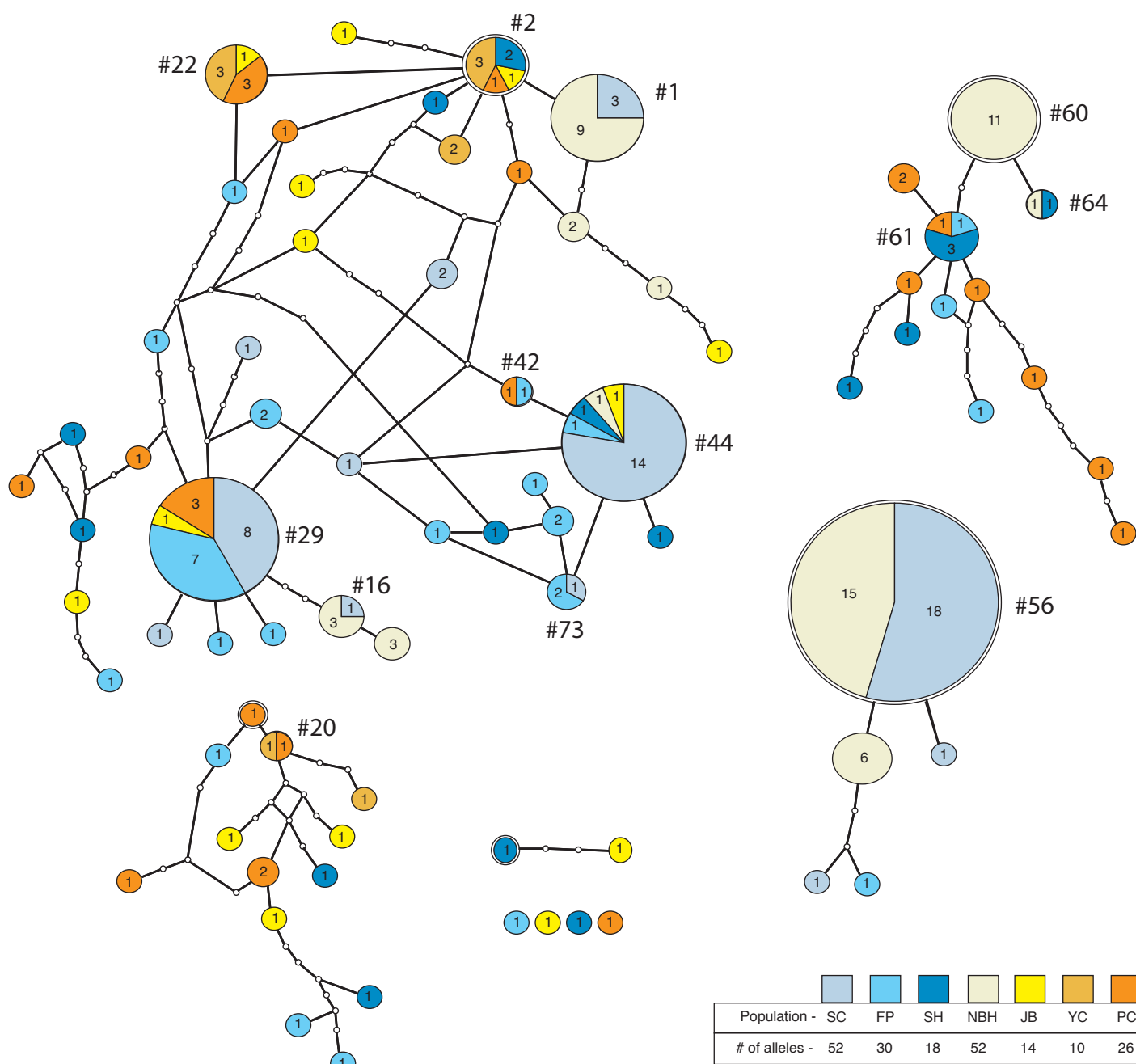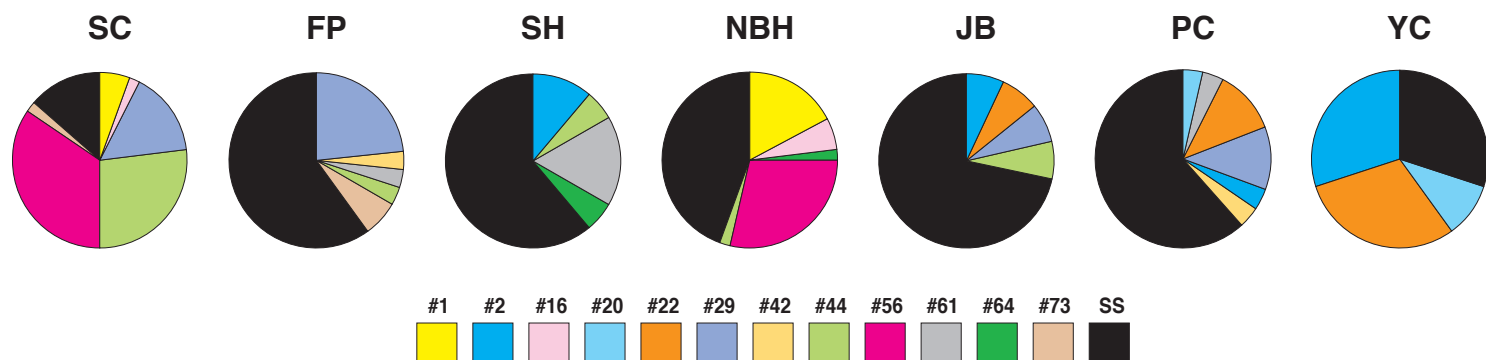

Supplement: Additional file 1: Figure S1 — AHR1 haplotype frequencies and distributions. Haplotypes were reconstructed using PHASE and genealogical relationships among the haplotypes were estimated using TCS software, as described in Materials and Methods. In the top panel, circles refer to unique haplotypes, wedges are colored by sampled population, and numbers within wedges refer to the number of alleles with that haplotype in the population represented by that color. Numbers outside of the circles refer to the haplotype number as shown in the bottom panel. In the bottom panel, black wedges indicate the percentage of site-specific (SS) haplotypes at each site and colored wedges indicate haplotypes shared among populations. [file 1471-2148-14-6-S1.pdf]

# AHR2 SNP Haplotype Distribution

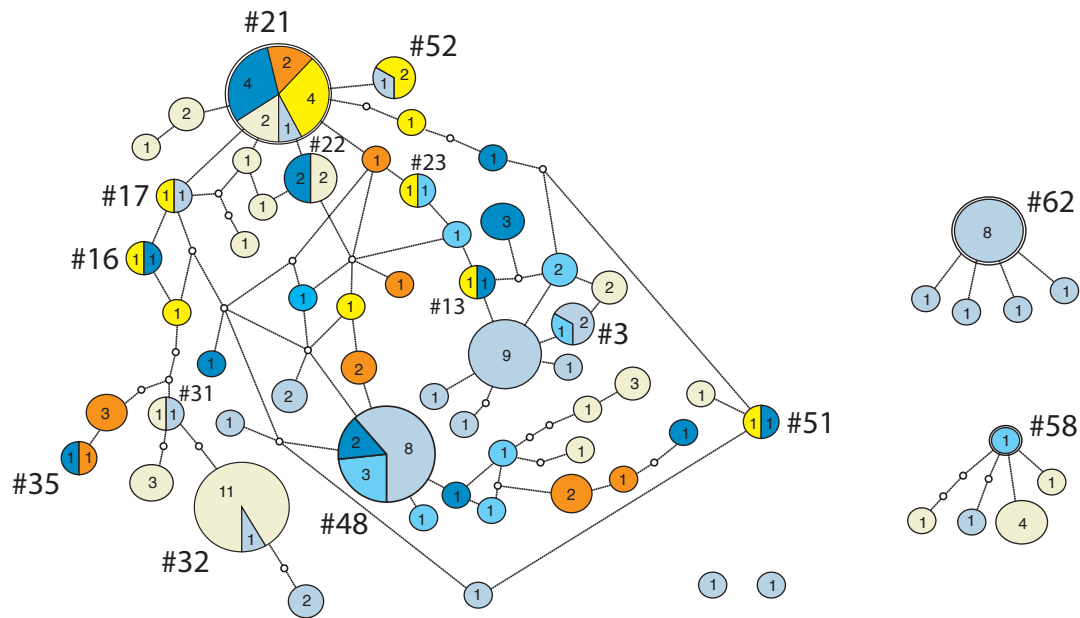

| Population -   | SC | FP | SH | NBH | JB | PC |
|----------------|----|----|----|-----|----|----|
| # of alleles - | 48 | 14 | 18 | 38  | 14 | 14 |

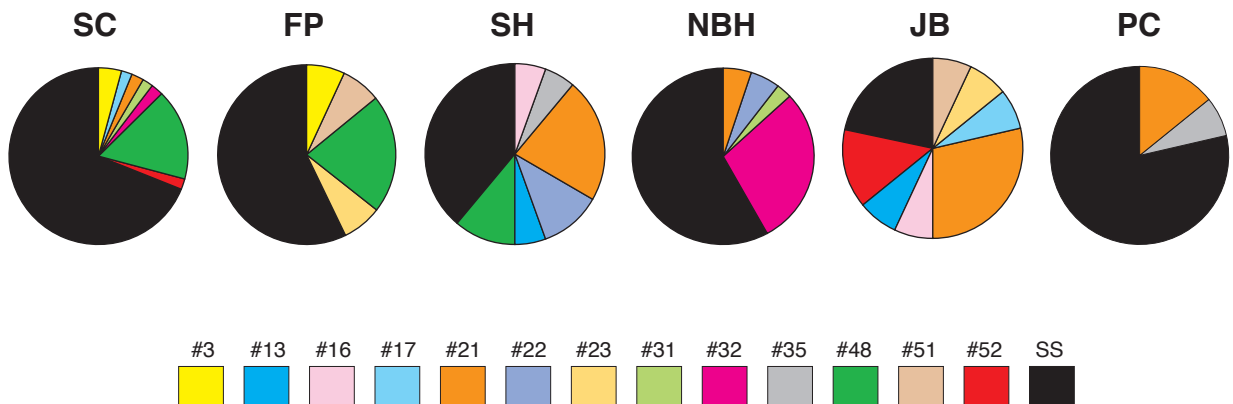

Supplement: Additional file 2: Figure S2 — AHR2 haplotype frequencies and distributions. Haplotypes were reconstructed using PHASE and genealogical relationships among the haplotypes were estimated using TCS software, as described in Materials and Methods. For additional description, see legend to Additional file 1: Figure S1. [file 1471-2148-14-6-S2.pdf]

# AHRR SNP Haplotype Distribution

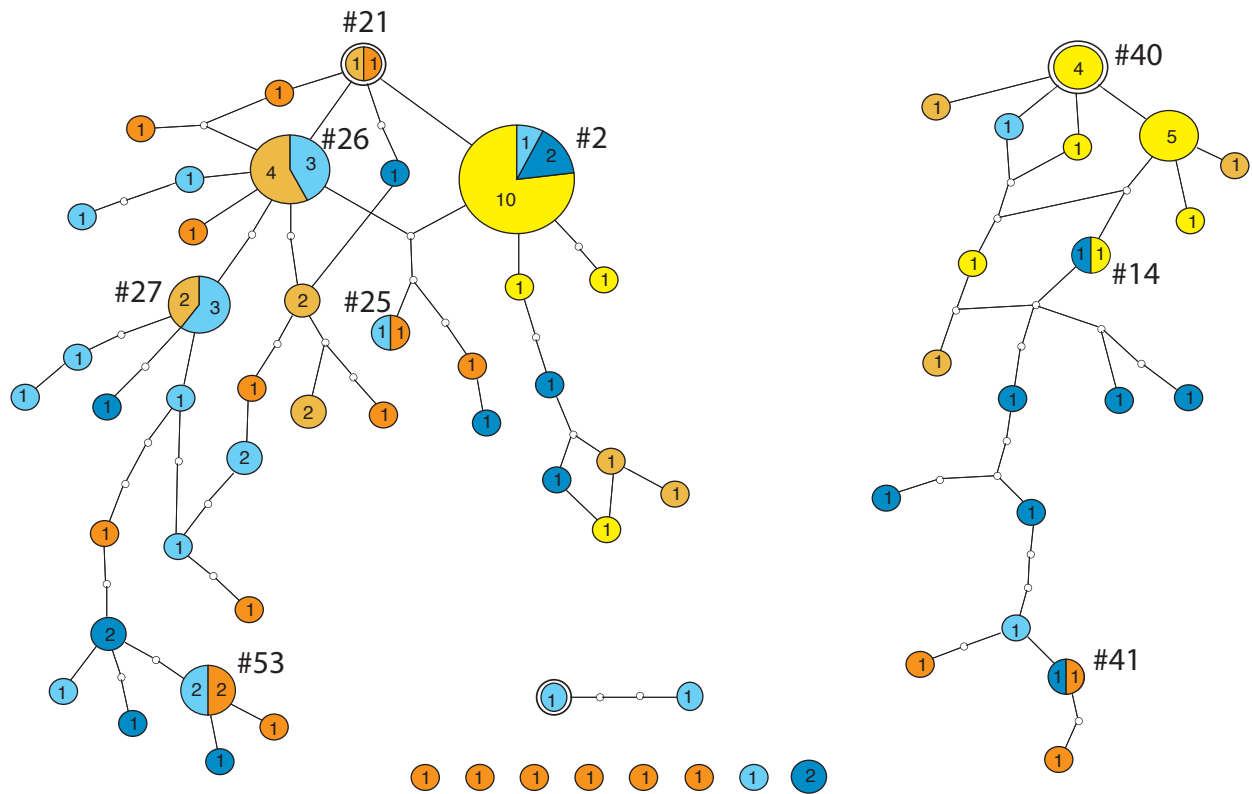

| Population -   | FP | SH | JB | YC | PC |
|----------------|----|----|----|----|----|
| # of alleles - | 24 | 20 | 26 | 16 | 22 |

FP

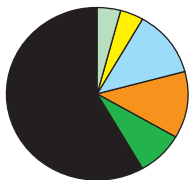

SH

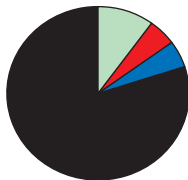

JB

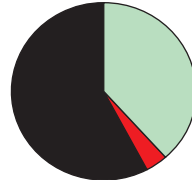

YC

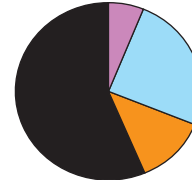

PC

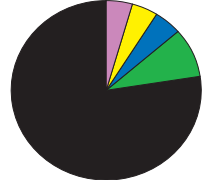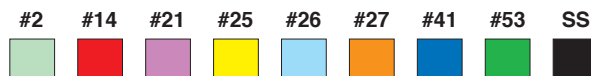

Supplement: Additional file 3: Figure S3 — AHRR haplotype frequencies and distributions. Haplotypes were reconstructed using PHASE and genealogical relationships among the haplotypes were estimated using TCS software, as described in Materials and Methods. For additional description, see legend to Additional file 1: Figure S1. [file 1471-2148-14-6-S3.pdf]
