# Supplementary material for: Genetic variation at aryl hydrocarbon receptor (AHR) loci in populations of Atlantic killifish (Fundulus heteroclitus) inhabiting polluted and reference habitats
Source: BMC Evol Biol. 2014 Jan 14;14:6. doi: 10.1186/1471-2148-14-6 (PMC3899389; doi:10.1186/1471-2148-14-6)
Supplement: Additional file 6: Table S3 — PCR primers. [file 1471-2148-14-6-S6.docx]

**Table S3.** PCR primers used in this study.

| Gene | Amplification primers | Sequencing primers |
| --- | --- | --- |
| *AHR1* | 5’ fragment (1): 1-28F–CCAAACGACATCGGGACCGGCTGAATGG  1-ov1r-CCTGGCCTTCCAAGGTAAGGGAATC  3’ fragment (2): 1-961R-CTCCAGTCATTTCTGCTTCCTCCAGTCC  1-ov2f-GGATCAGTCTGTATATGTCTGCCAACC | 5’ fragment (1): 1-ov1a -GAGATGGAACCTGACAGCAGCTCTTC  1-ov1b -GTGTTCAGGCTCCTCACTAAAGAG  1-ov1c -CTCTGGGTGGCGATGATGTAGTCC  1-ov1d -CTCTCCAGGAAGGAGGAATTCTCTGG  3’ fragment (2): 1-ov2g -CACCGTCTGAAACTAATCCCAGTC -2038)  1-ov2h -CGGGTCCACAGATTTTGGTTTAG  1-ov2i -GGAAGGTCTGTCATTCTGTAGTCGG -2597)  1-ov2j -GATAGGAGCCTGCCCCACCAGATGAG -2103) |
| *AHR2* | 2-5UTR- GGACGGAAGCTGGCGGAGGACTGAGAAG  2-949R- GGGCAGGACTGATGAAAAGC  2-969R* -AAACTATGCTGAGGTTGGGTCCTCCTGC  *used in NBH and SC samples only | 2-226F -CCACGGGCGACTAAAGTACC  2-446F -GCAACGAGTGGCAAGAGAGCAA  2-726F –AAGTTCTGCTCCTCATATGAGC  2-5UTR- GGACGGAAGCTGGCGGAGGACTGAGAAG  2-949R- GGGCAGGACTGATGAAAAGC  2-750R –TATGGGAACTCTTGGGTGGC  2-502R -CGATCACAGCTACCACTGAC  2-262R –ATGGCGATGGCAAACAGAGC  2-40NF* -CGTACGCCAACAAGAAGAGGAA |
| *AHRR* | RRterm3-CATTTATCTGCCCATGCGTGGGTAATGCTCTC  RRkoz3-GGATATTCTGGCGCACCTGCTACCTCC  RR242- AAAGTGGATGTGATTCTGGAGC  RR2418-GGCGGGGCATACATTTATCTGC | RRterm3-CATTTATCTGCCCATGCGTGGGTAATGCTCTC  RRkoz3-GGATATTCTGGCGCACCTGCTACCTCC  RRa-CATCCACATAGACGACCGTCAGGAG  RRb-GAGGAATGGGTGACATGACAGACC  RRc-GATCGACCGGCGCTACGGTAACGG  RRd-GGTCGTACACCCCGTTACCGTAG  RRe-GGTCTGTCATGTCACCCATTCCTC  RRf-CTGATGAAATCCTAGATAGTCCAC  RR242- AAAGTGGATGTGATTCTGGAGC  RR2418-GGCGGGGCATACATTTATCTGC |
